# A light in the shadow: the use of Lucifer Yellow technique to demonstrate nectar reabsorption

**DOI:** 10.1186/1746-4811-9-20

**Published:** 2013-06-19

**Authors:** Poliana Cardoso-Gustavson, João Marcelo Robazzi Bignelli Valente Aguiar, Emerson Ricardo Pansarin, Fábio de Barros

**Affiliations:** 1 Programa de Pós-Graduação em Biodiversidade Vegetal e Meio Ambiente, Instituto de Botânica, C. Postal 68041, São Paulo, SP, Brazil; 2 Programa de Pós-Graduação em Biologia Comparada, Faculdade de Filosofia, Ciências e Letras de Ribeirão Preto, Universidade de São Paulo, Av. Bandeirantes 3900, 14040-901, Ribeirão Preto, SP, Brazil; 3 Departamento de Biologia, Faculdade de Filosofia, Ciências e Letras de Ribeirão Preto, Universidade de São Paulo, Av. Bandeirantes 3900, 14040-901, Ribeirão Preto, SP, Brazil; 4 Núcleo de Pesquisa Orquidário do Estado, Instituto de Botânica, C. Postal 68041, 04045-972, São Paulo, SP, Brazil

**Keywords:** *Cucurbita Pepo*, Fluorescence Microscopy, LYCH, Nectar Pathway, *Passiflora Edulis*

## Abstract

**Background:**

Nectar reabsorption is a widely known phenomenon, related to the strategy of resource-recovery and also to maintain the nectar homeostasis at the nectary. The method currently performed to demonstrate nectar being reabsorbed involves the use of radioactive tracers applied to the nectary. Although this method works perfectly, it is complex and requires specific supplies and equipment. Therefore, here we propose an efficient method to obtain a visual demonstration of nectar reabsorption, adapting the use of Lucifer Yellow CH (LYCH), a fluorescent membrane-impermeable dye that can enter the vacuole by endocytosis.

**Results:**

We applied a LYCH solution to the floral nectary (FN) of *Cucurbita pepo* L., which is a species known for its ability of nectar reabsorption, and to the extrafloral nectary (EFN) of *Passiflora edulis* Sims which does not reabsorb the secreted nectar. In all tests performed, we observed that LYCH stained the nectary tissues differentially according to the reabsorption ability of the nectary. The treated FN of *C. pepo* presented a concentrated fluorescence at the epidermis that decreased at the deeper nectary parenchyma, until reaching the vascular bundles, indicating nectar reabsorption in the flowers of the species. In contrast, treated EFN of *P. edulis* presented fluorescence only at the cuticle surface, indicating that nectar is not reabsorbed by that particular tissue.

**Conclusion:**

LYCH is an efficient marker to demonstrate nectar reabsorption.

## Background

Nectar reabsorption is a phenomenon known for many plant families, occurring as a resource-recovery strategy and also in order to maintain the nectar homeostasis at the nectary. Several direct and indirect demonstrations of nectar reabsorption have been published [see reviews in [1,2], highlighting the micro-radiography technique in which ^14^C-labelled sucrose is incorporated by nectaries making it possible to follow the detailed rate of movement of radioactive tracer within the nectary and in other plant tissues [2] and references therein]. This technique, however, is complex and requires some specific laboratory equipment and hazardous supplies.

The substitution of floral nectar by a dilute solution of vital stains (e.g. neutral red) is not recommended, since vital dyes diffuse across the tissues without specificity. Although Schiff’s reagent bonds to 1,2 – glicol (after a pretreatment with periodic acid), it shows a weak reaction with glucose and sucrose even in an analysis with purified sugars [3], being also not adequate to specifically track nectar itself through the plant tissues. In other words, to find a dye that can efficiently mark reabsorbed nectar in plant tissues, it is necessary that it somehow interacts with sugar molecules present within nectar itself and moves with them, signalizing the complete pathway.

Lucifer Yellow CH (LYCH), which is 6-amino-2,3-dihydro-1,3-dioxo-2-hydrazinocarbonylamino-1H-benz[d,e]isoquinoline-5,8-disulfonic-acid dilithium salt, and other fluorescent apoplastic tracers have been used to demonstrate the route of the absorbed water present in air humidity by the foliar epidermis [4-6]. These experiments were developed in a passive way, applying a solution of LYCH over the leaf surface and submitting the plant to dry conditions and later it was possible to visualize the apoplastic route of the absorbed solution. LYCH has been also extensively used as fluorescent tracer in studies of intercellular communication in plants and relationships between sucrose and phloem [7-9], since it is transported in parallel with sucrose, and can accumulate in the vacuole. An evidence of the interaction between LYCH and sucrose is that the uptake of the external dye marker occurs preferentially in the presence of this sugar [9,10]. This tracer has also been applied in studies of the process of nectar secretion [11,12]. In those studies, LYCH was injected into the nectary or nectar cells, forcing the dye to be incorporated by the plant.

The properties of LYCH can bring several advantages for the use as a marker for nectar reabsorption: a very high quantum yield resulting in easily visualized fluorescence after uptake by cells; small size of the LYCH fluorescent molecule, allowing passage through the cell wall; stability over a wide range of pH, from 2.0-10.0; and absence of toxicity to cells [7,13-15]. Considering that the reabsorption of nectar may occur through the apoplast or symplast pathways [16], the characteristics of LYCH allow tracing in these both pathways.

Our intention was to find a reliable and simpler method to visually demonstrate the nectar reabsorption phenomenon, in the most faithful way to what happens naturally. For that, we performed tests with LYCH dye, adapting its already known use in studies of nectar secretion and nectary structure [11,12], on the floral nectaries of *Cucurbita pepo* L. (Cucurbitaceae), a species recognized by its ability for nectar reabsorption [2,16,17], and extrafloral nectaries of *Passiflora edulis* Sims (Passifloraceae), where structural characteristics indicates that nectar reabsorption does not occur [18,19].

## Results and discussion

In all tests performed it is unequivocal that LYCH marks the pathway of nectar according to the ability of the nectary to reabsorb it. LYCH does not spread to tissues during fixation; this implies that the movement of LYCH throughout the cytoplasm is due to the way in which it is taken into the cell and not to the fixation process [13,20]. The reaction of LYCH with fixative clearly avoids several problems associated with the localization of water-soluble dyes in plant tissues [21].

### Reabsorption process is visualized on FN in male flowers of *Cucurbita pepo* L

**Figure 1:**
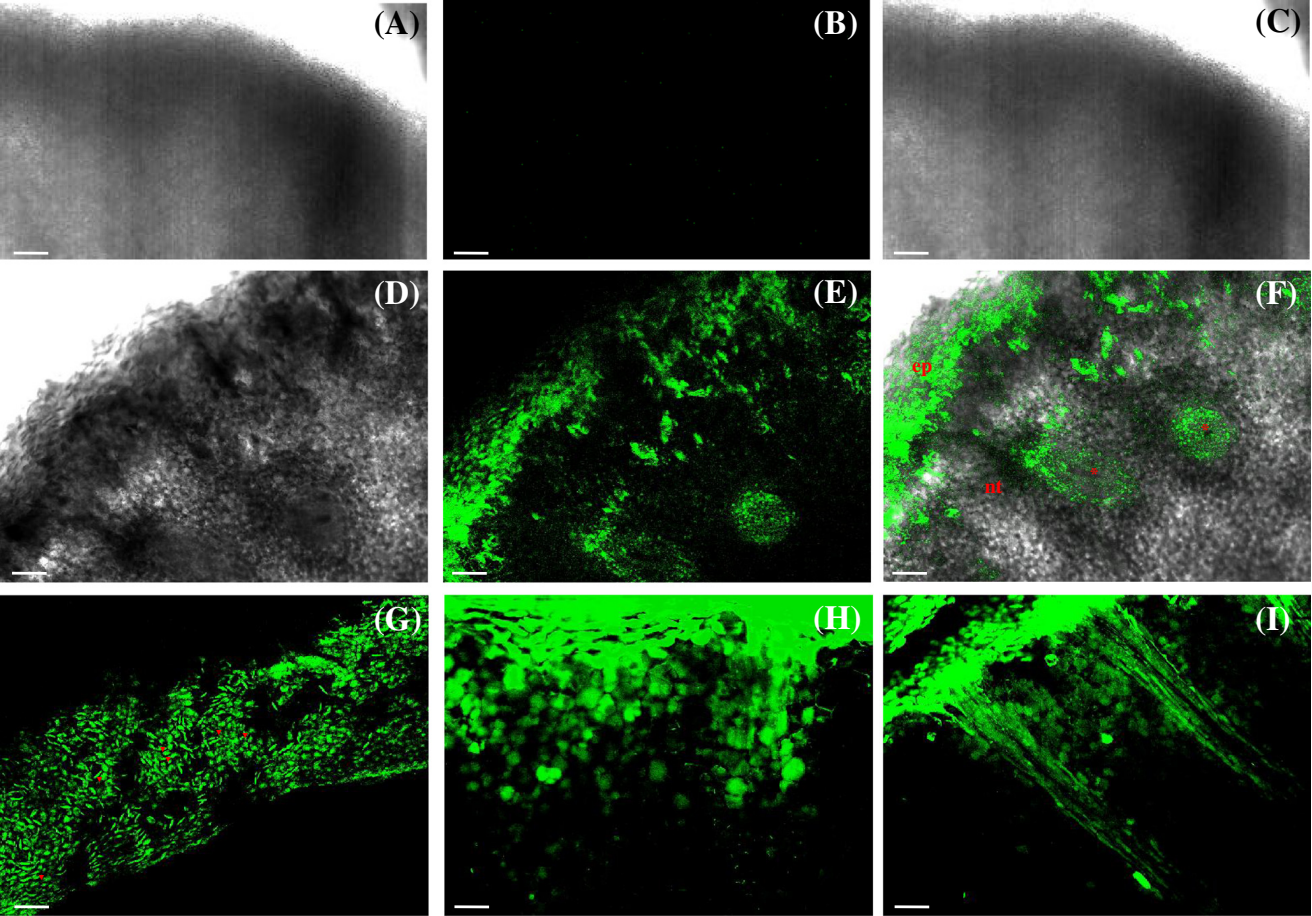
**LYCH dye efficiently stains the reabsorbed nectar pathway through nectary tissues.** Hand sections of floral nectaries (FN) of *Cucurbita pepo* 24 h after the introduction of the LYCH solution onto nectar standing at the flower base. **(A, D)** Transmission image. **(B, E, G, H)** LYCH fluorescence (485 nm excitation, 530 nm emission). **(C, F, I)** Overlay projection images of transmission and fluorescence. **(A-C)** FN treated with distillated water instead of LYCH solution. Note the absence of fluorescence (B,C). **(D-I)** Different regions of the same nectary: labelled cells in epidermis (ep) **(E-G)**, nectary tissue (nt), inner parenchyma and vascular bundles (*) **(F, H-I). (G)** Epidermis in frontal view; arrowhead, stomata. Scale bars: 25 μm.

Control treatment showed no fluorescence (Figure 1A-C). A very strong fluorescence is observed within the nectary epidermis illustrated in frontal view (Figure 1G) indicating the presence of LYCH over it. The structural description of the FN indicates that nectar exudes from the stomata and forms a continuous layer on the nectary surface [22]. LYCH fluorescence was concentrated in the epidermis and in the nectary parenchyma layers, decreasing in the deeper part of the parenchyma, reaching the vascular bundles (Figure 1E-I). This experiment revealed the same result reported with the use of ^14^C-sucrose [17], confirming the use of LYCH as indicative for nectar reabsorption.

According to ultrastructural studies of nectar reabsorption in *Platanthera chlorantha*[23], numerous vesicles are associated with the plasmalemma during the stage of reabsorption, and it is likely that they are formed by endocytosis. Turgor pressure may prevent endocytosis, but evidence from studies on the uptake of membrane-impermeable molecules support the operation of endocytosis in plants and argue for the hypothesis that vesicles are produced by this process in *P. chlorantha*[23] and references therein]. Considering the properties of LYCH as a fluorescent membrane-impermeable dye, it is plausible to use it as a marker for nectar reabsorption.

### No reabsorption process is observed on EFN of *Passiflora edulis* Sims

**Figure 2:**
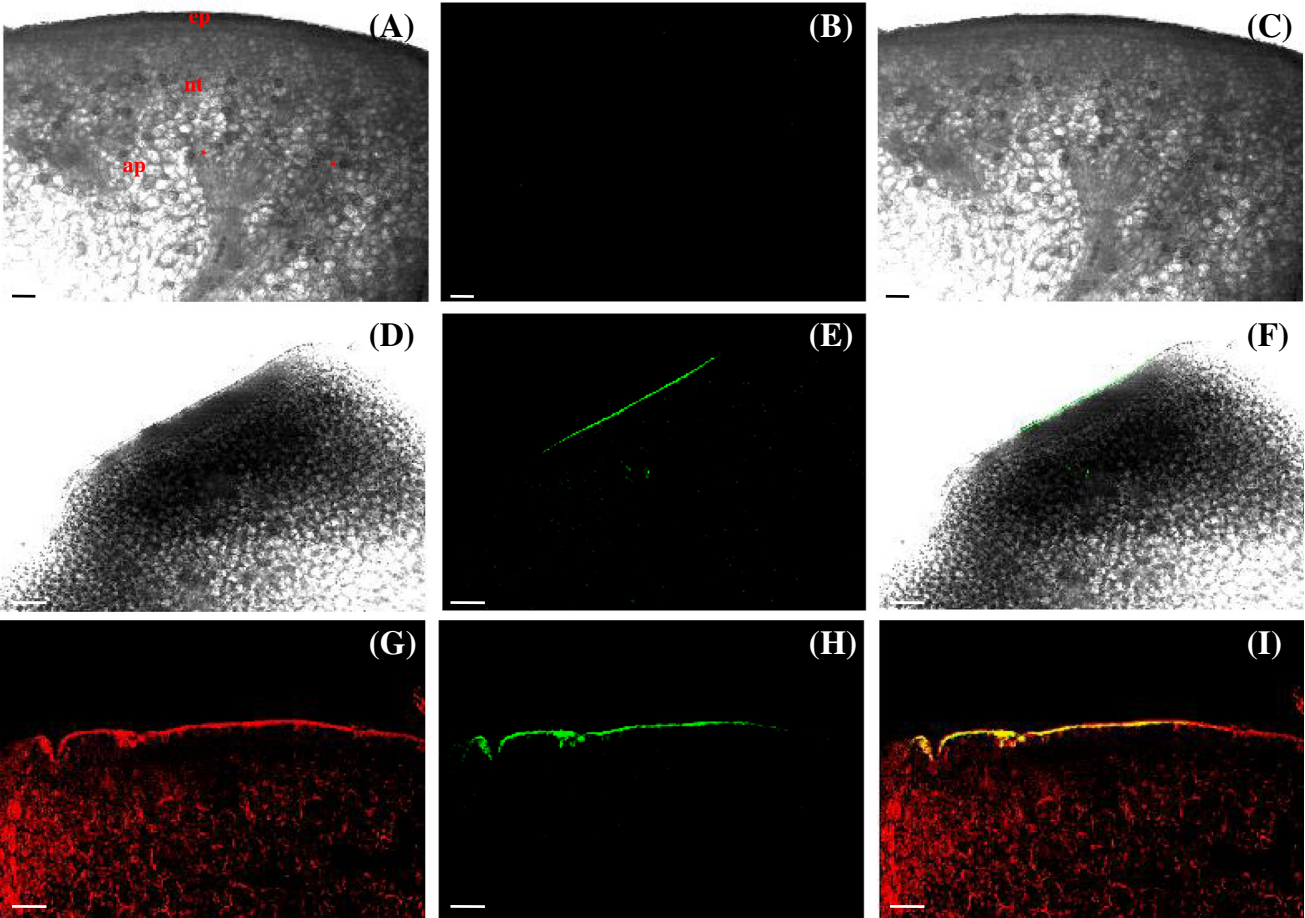
**LYCH dye moves with the nectar.** Hand sections of extrafloral nectary (EFN) of *Passiflora edulis* 24 h after the application of the LYCH solution. **(A, D, G)** Transmission image. **(B, E, H)** LYCH fluorescence (485 nm excitation, 530 nm emission). **(C, F, I)** Overlay projection images of transmission and fluorescence. **(A-C)** EFN treated with distillated water. Note the absence of fluorescence; ep, epidermis; nt, nectar tissue; ap, adjacent parenchyma, *, vascular terminations. **(E-I)** Cuticle labelled. **(G-I)** Live EFN. **(G)** Autofluorescence of the cuticle and chloroplasts present in nectary tissue and adjacent parenchyma. **(H, I)** Positive result in the cuticle when only the emission of LYCH is selected. Nectar traces remain attached to the cuticle and cuticular flanges. Scale bars: 25 μm.

The tests with LYCH were positive only in some portions of the cuticle and cuticular flanges, indicating that there was no influx of nectar into the nectary (Figure 2E-I). We supposed that the aqueous portion of the nectar evaporated, leaving the sugar and LYCH over the EFN surface. Besides fixing the EFN, we also carried out the treatment with LYCH in fresh glands. It was possible to visualize the autofluorescence of the cuticle and chloroplasts (Figure 2G). After the selection of the LYCH-emission wavelength, we were able to visualize the same results observed in fixed material (Figure 2H-I). To avoid the interference of autofluorescence in results from fresh material, we strongly recommend the use of aldehyde-fixatives, especially if the visualization is under epifluorescence microscope, in which only the LYCH-emission wavelength cannot be selected. The control treatment showed no fluorescence (Figure 2A-C).

Since nectar of *Passiflora edulis* possesses glucose, fructose and sucrose [19], we associate the non-occurrence of reabsorption in EFN of *Passiflora* to the presence of a relative thick cuticle and cuticular flanges. The abundance of wax can vary widely and is an important factor for permeability: a cuticle rich in wax favors the passage of fat soluble substances but is a significant barrier for water soluble compounds [24]. There is probably little or no wax in the cuticle of nectaries of *Cucurbita pepo*, which also enables some nectar to emerge through ruptures or openings in the cuticle [22].

Unlike studies where LYCH was applied into the nectaries [11,12], we tried to keep the phenomenon of nectar reabsorption away from any surrounding interference, using the inert fluorescent dye to passively follow the natural pathway of the reabsorbed nectar in the tissues (or retained outside the gland, like at the *P. edulis* EFN) without cutting the flower parts or injecting LYCH directly into the tissues. That is also the reason why we applied the solution of LYCH directly over the nectar, unlike what is performed when applying radioactive labeled techniques, where the nectar is removed and replaced by the marked artificial nectar [16]. Thus, our experiment was performed in order to preserve as much as possible the structure of the nectary, maintaining its natural capacity of reabsorption. Here, we demonstrate the efficiency on the use of LYCH as a fluorescent tracer to observe nectar reabsorption by contrasting glands that reabsorb/not reabsorb to emphasize that this is a trustful technique. Nectar reabsorption is generally accepted as a commonly-occurring phenomenon although the actual process of nectar reabsorption is understudied [2]. Hence, this paper provides a method toward visualization of this process, hoping it can help on future studies that aim to clear the cytological mechanisms of nectar reabsorption.

## Methods

### Plant material

#### Floral nectaries (FN) of male flowers of Cucurbita pepo *L*

**Figure 3:**
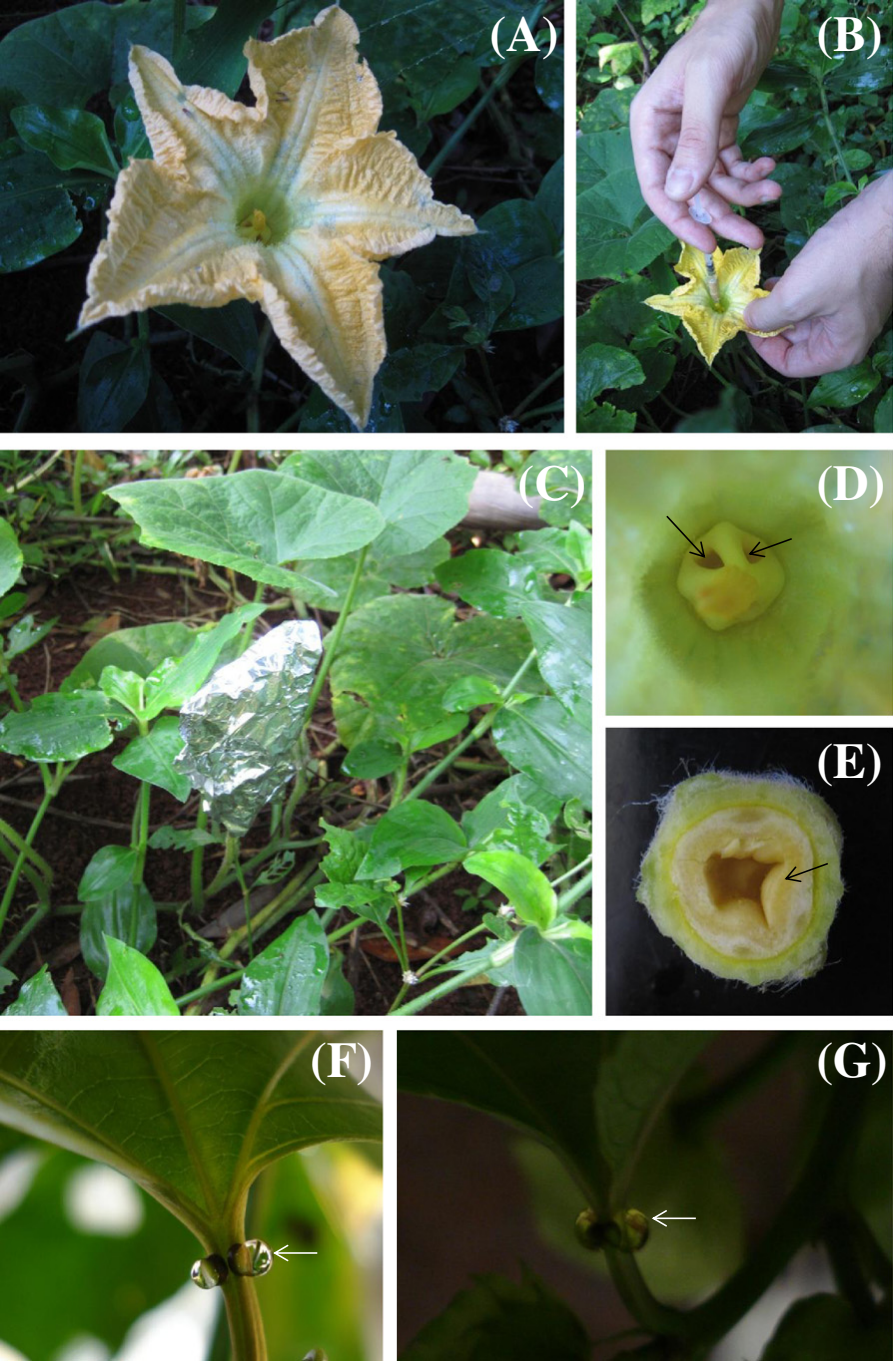
**Field procedures and morphological aspects of the nectaries. (A)** Floral nectary of a male flower of *Cucurbita pepo***(B)** Application of 0.3 ml of 1% LYCH onto the accumulated floral nectar. **(C)** Flower immediately wrapped with aluminum foil to avoid sunlight. **(D)** Detail of the androecium. Note the pores of the nectary (arrows). **(E)** Transversal section of the nectary. Note the nectary tissues (arrow). **(F, G)** Extrafloral nectaries (EFN) in the petiole of *Passiflora edulis* Sims. **(F)** Nectar over the surface of the EFN (arrow). **(G)** Plant kept in the dark immediately after the application of a drop of LYCH over the nectar. Note the yellow aspect of the drop after the LYCH application (arrow).

Flowers from three plants cultivated in the greenhouse of “Faculdade de Filosofia, Ciências e Letras de Ribeirão Preto” from the University of São Paulo were tested (Figure 3A). Nectar of *C. pepo* is sucrose dominant, regardless of the sex and age of the flower [17]. Relevant morphological and structural aspects [16,22] are detailed: the nectary can be found at a cavity in the base of the partially fused filaments and is accessible through pores of the nectary (Figure 3D). The nectary is composed of an epidermis with stomata from which nectar exudes, and a nectar producing parenchyma (Figure 3E) that receives xylem vessels and phloem sieve tubes.

#### Extrafloral nectaries (EFN) of Passiflora edulis Sims

Nectar glands present on petioles from second and third visible nodes of three individuals cultivated at the Institute of Botany from São Paulo were tested. The base of branches was isolated with Tanglefood® grease to avoid the removal of the nectar by ants or other insects. These glands exude profuse nectar available during daytime (Figure 3F). The nectar of *P. edulis* is composed by sucrose, glucose and fructose [19]. Some relevant anatomical details are pertinent [19]: the multiple epidermis is covered by a cuticle and cuticular flanges extend up to the inner periclinal wall of the first cell layer. The nectar rising out of the epidermis promotes the cuticle detachment, although cuticle disruption is observed only when ants have access to the EFN. Nectary tissue and the adjacent parenchyma are distinct and separate from each other by vascular terminations.

### Field and laboratory procedures

A 1% (w/v) solution of Lucifer Yellow CH dilithium salt (Sigma-Aldrich) in distilled water was prepared in the dark at room temperature.

In the morning (ca. 10 am), 0.3 ml of the solution was applied to the nectary of freshly opened male flowers of *C. pepo* (Figure 3B). Note that no previous manipulations were performed and the stain was mixed to the available floral nectar. The flowers were immediately covered with an aluminum foil (Figure 3C) and kept in the dark during 24 h. In the subsequent morning, the androecium (Figure 3D) of each flower was removed and the FN was isolated (Figure 3E). Nectar glands were washed several times with distilled water and immediately fixed with Karnovsky’s glutaraldehyde solution [25] for 24 h. After the fixation the material was washed several times in distilled water, free-hand sectioned and slides mounted in water.

A drop of LYCH was applied over the nectar of each EFN of *Passiflora edulis* (Figure 3G) in the morning (9 am) and the vases were kept in the dark during 24 h. The same procedure was performed with treated flowers of *Cucurbita pepo.* Also, we avoided the fixation step for a pair of glands to compare the results from fresh material to fixed ones. All glands (fixed and fresh) were free-hand sectioned and slides mounted in water.

Control treatments were carried out simultaneously to each LYCH test, submitting FN and EFN to the same procedures with distilled water (Figures 1A-C and 2A-C).

A Zeiss LSM 510-Meta laser scanning confocal microscope was used to obtain the images of the LYCH stained tissues and controls. LYCH was excited by 488 nm and a 530 nm emission filter. In some instances, the tissue was photographed using a combination of emitted and transmitted lights. The resultant images could be overlaid to determine the location of the fluorescent signal.

## Abbreviations

LYCH: Lucifer yellow dye; FN: Floral nectaries; EFN: Extrafloral nectaries.

## Competing interests

The authors declare that they have no competing interests.

## Authors’ contributions

PCG planned the research, carried out the techniques, and drafted the manuscript. JMRBVA also participated in the techniques and in the manuscript writing. ERP and FB contributed with critical review of the manuscript, provided lab support and orientation during the development of this work. All authors read and approved the final manuscript.
